# Farmer and Veterinarian Attitudes towards the Bovine Tuberculosis Eradication Programme in Spain: What Is Going on in the Field?

**DOI:** 10.3389/fvets.2017.00202

**Published:** 2017-11-27

**Authors:** Giovanna Ciaravino, Patricia Ibarra, Ester Casal, Sergi Lopez, Josep Espluga, Jordi Casal, Sebastian Napp, Alberto Allepuz

**Affiliations:** ^1^Departament de Sanitat i Anatomia Animals, Universitat Autònoma de Barcelona (UAB), Barcelona, Spain; ^2^ASANA (Asociación Andaluza de Antropología), Sevilla, Spain; ^3^Departament de Sociologia/IGOP—GEPS—ARAG-UAB, Universitat Autònoma de Barcelona (UAB), Barcelona, Spain; ^4^Centre de Recerca en Sanitat Animal (CReSA)—Institut de Recerca i Tecnologia Agroalimentàries (IRTA), Campus de la Universitat Autònoma de Barcelona, Barcelona, Spain

**Keywords:** bovine tuberculosis, qualitative epidemiology, ethnography, sociological factors, disease eradication

## Abstract

The effectiveness of health interventions against bovine tuberculosis (bTB) is influenced by several “*non-biological*” factors that may hamper bTB detection and control. Although the engagement of stakeholders is a key factor for the eradication programme’s success, social factors have been often ignored in the control programmes of animal diseases, especially in developed countries. In this study, we used a qualitative approach to investigate perceptions, opinions, attitudes, and beliefs of farmers, and veterinarians who may influence the effectiveness of the Spanish bTB eradication programme. The study was carried out in two phases. First, 13 key representatives of different groups involved in the programme were interviewed through exploratory interviews to identify most relevant themes circulating in the population. Interviews focused on strong and weak points of the programme; reasons for failure to achieve eradication; benefits of being disease free; future perspectives, and proposed changes to the programme. Based on these results, a thematic guide was developed and detailed information was gained through face-to-face in-depth interviews conducted on a purposive sample of 39 farmers and veterinarians. Data were analysed following an ethnographic methodology. Main results suggested that the bTB programme is perceived as a law enforcement duty without an adequate motivation of some stakeholders and a general feeling of distrust arose. The complexity of bTB epidemiology combined with gaps in knowledge and weak communication throughout stakeholders contributed to causing disbeliefs, which in turn generated different kinds of guesses and interpretations. Low reliability in the routine skin test for bTB screening was expressed and the level of confidence on test results interpretation was linked with skills and experience of public and private veterinarians in the field. Lack of training for farmers and pressure faced by veterinarians during field activities also emerged. Few benefits of being bTB free were perceived and comparative grievances referred to wildlife and other domestic reservoirs, sector-specific legislation for bullfighting farms, and the absence of specific health legislation for game hunting farms were reported. Understanding reasons for demotivation and scepticism may help institutions to ensure stakeholders’ collaboration and increase the acceptability of control measures leading to an earlier achievement of eradication.

## Introduction

The influence of social factors on public health interventions is well known in human medicine and several studies taking these aspects into account have been done (1–3); however, these aspects have been often ignored in the implementation of animal health programmes. Recently, the situation has changed and the interest on the influence of social factors in the control programmes of animal diseases has greatly increased. As a matter of fact, several studies have highlighted the importance of understanding the attitudes and behaviours of the different stakeholders involved, as their actions have a great influence on the effectiveness and sustainability of such programmes (4–9).

The use of participatory approaches to investigate attitudes and behaviours is a valuable tool to conduct such studies (5). The fundamental principle of participatory research is that emphasises “*knowledge for action*” and a “*bottom up approach*” in contrast to conventional research, which is more “*top-down*” (10). The use of such approaches provides a voice to the different stakeholders increasing, in that way, the understanding of health problems and the options for their prevention, control, and surveillance (11).

In the last years, different qualitative methods, such as semi-structured interviews, focus group discussions, ranking and scoring methods, or visualisation and diagramming, have been used in the field of Veterinary Medicine (5). The increased interest in these approaches has been reflected in an increase in participatory epidemiology activities in animal health, especially from 2012; however, most of them have been implemented in Asia and Africa but not so much in Europe (12).

The engagement of stakeholders and the level of acceptability of the interventions are key factors for the success of control programmes and surveillance systems (13). The application of qualitative methods can ensure the access to specific type of information and local knowledge otherwise impossible to collect; it can contribute to identifying information gaps, understanding local cultures and beliefs, and setting priorities (11, 14). Moreover, it allows investigating risk perception amongst stakeholders and the impact it may have on their response and commitment towards health policies. Finally, since the application of qualitative methods results in a high level of community participation throughout the decision process of designing health interventions, it ensures a more accurate implementation and helps in developing good relationships with communities and in reducing later conflicts.

Bovine tuberculosis (bTB) in Europe represents a significant obstacle to the sustainability of the livestock sector and since 1964 many efforts have been made to eradicate it (15). Even though, substantial improvement in the prevalence reduction has been achieved, the eradication of bTB remains a challenge. While in some countries, such as Germany, The Netherlands, and Belgium, the eradication campaigns have been successful; in other countries, such as the United Kingdom, Ireland, Italy, and Spain, the disease is still endemic. Furthermore, recently the re-emergency of the disease in officially bTB free countries has been reported (16).

In Spain, several aspects of bTB epidemiology have been investigated. In particular, research has been conducted on: spatial and spatiotemporal dynamics of the disease (17–19); risk factors associated with bTB persistence and new infections in cattle herds (20–22); the role of wildlife reservoirs (23–31) and the role of other domestic reservoirs (32, 33).

In spite of all these studies, no major decrease in the bTB herd prevalence has been observed in Spain over the last decade (1.8% in 2004 and 1.7% in 2014) and, in 2015, the bTB prevalence has increased to 2.8% (34). This context makes it necessary to study other factors that might influence the success of the national bTB eradication programme, such as sociological and anthropological factors that have never been central in such investigations.

In this study, we aim to investigate farmers and veterinarians’ perceptions, opinions, attitudes, and beliefs about the Spanish bTB eradication programme by using a qualitative approach in order to assess the influence that these aspects may have on the effectiveness of the programme.

## Materials and Methods

### Study Areas

**Figure 1:**
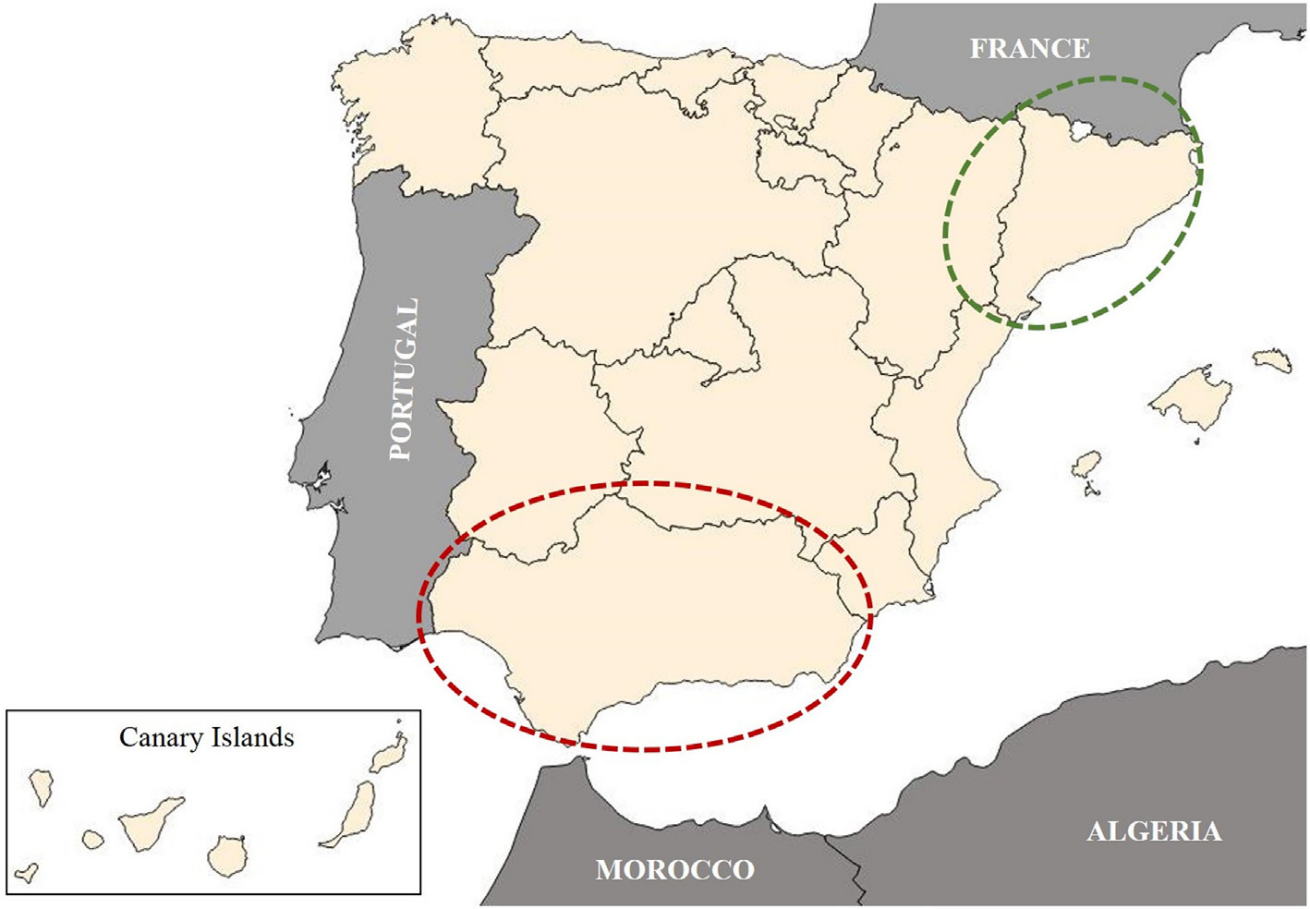
Map of Spain by Autonomous Communities is shown in the figure. Dotted ellipses indicate the two study areas. Red dotted ellipses: Andalusia, high prevalence area. Green dotted ellipses: Catalonia, low prevalence area. The Canary Islands, an Autonomous Community of Spain located in the Atlantic Ocean (west of Morocco), are illustrated in box at the bottom-left corner of the map.

The study was carried out in two Autonomous Communities of Spain, Andalusia and Catalonia, as representatives of high- and low-prevalence areas, respectively (Figure 1).

In Spain, Regional Veterinary Services (RVS) has been set up in each Autonomous Community under the coordination of the Spanish Ministry of Agriculture and Fisheries, Food and Environment. Moreover, each administrative county has a Local Veterinary Service (LVS) attached to the RVS. Besides, there are accredited veterinarians working in the field (private sector) that collaborate in carrying out disease prevention programmes. Often, they are also responsible for hygiene, productivity, and treatment programmes of the same farms.

#### Catalonia

Catalonia is located on the north-eastern extremity of the Iberian Peninsula; it consists of 4 provinces and 42 counties. The Autonomous Community can count on 47 official veterinarians working on bTB at the LVS and 113 specialised private veterinarians supporting the routine screening tests for bTB in about 1,900 beef herds, 700 dairy herds, and a few bullfighting herds. Since 2008, the bTB herd prevalence at regional level remained lower than 1%, decreasing to 0.04% in 2013, but in 2015 bTB herd prevalence slightly increased to 0.32% (34).

#### Andalusia

Andalusia is located in southern Spain and it is divided into 8 provinces and 62 counties. There are 63 official veterinarians, operating at the LVS, directly engaged with the bTB eradication programme. These are assisted by about 270 specialised private veterinarians for the implementation of routine screening in about 5,300 beef farms, 800 dairy farms, and 400 bullfighting farms. Over last 10 years, herd prevalence for bTB in this region has persisted above 4% and in the last 2 years has dramatically increased to 11% in 2014 and 17% in 2015 (34).

### Study Design

The present study was carried out in two phases, first, exploratory interviews followed by qualitative in-depth interviews, and it was conducted by a team of veterinarians, sociologists, and anthropologists. In both phases, people to be interviewed were selected through a purposive sampling.

In accordance with the national and institutional guidelines, ethical approval was not required for this study as it did not include samples or experiments on people but only their expression of opinions in relation to a specific topic.

With regard to the informed consent of participants: as the interviews were anonymous, the data were analysed anonymously and the decision to participate in the study was solely up to each contacted person, we did not consider it necessary to obtain a written consent. We orally informed all participants of the elements of consent and permission was obtained verbally before starting the interview.

At the beginning of each interview: interviewers introduced themselves and the contacted person was informed on the study design and its objectives.

It was explained that the participation was voluntary and completely anonymous (data collection and analysis) and that they could stop the interview at any time.

It was explained that there were no expected risks and no expected personal benefits associated with participation in the study. We also asked their approval for using information collected through the interview and for using direct quotes from them and these would only be cited as from a “farmer” or “veterinarian,” keeping the anonymity.

#### Exploratory Interviews

The aim of these interviews was to identify major themes to be considered and further investigated in the qualitative in-depth interviews. For that purpose, we used a *stakeholder sampling* strategy (35) by which we selected a particular segment of the population having concrete experience with the issue at stake (bTB) or being strongly affected by it. The concrete population segments were “farmers” and “veterinarians” of the study areas.

Overall, 13 key representatives were interviewed. In the high-prevalence area (Andalusia), three veterinarians of the public sector (one from the RVS, one from the LVS, and one from the diagnostic laboratory), two private veterinarians (operating in two different counties), and three farmers, covering the main livestock production types: beef, dairy, and bullfighting farms were interviewed. In the low-prevalence area (Catalonia), two veterinarians of the public sector (RVS and LVS), one private veterinarian, and two farmers (beef and dairy farmers) were also interviewed.

The interviews were conducted face-to-face following a general script in order to allow, as much as possible, open and informal conversations in which key aspects on the bTB management could emerge.

Each interview lasted between 50 and 120 min and focused on the following six points: (i) strong points of the bTB eradication program; (ii) weak points of the bTB eradication program; (iii) reasons for the failure of bTB eradication; (iv) future perspectives; (v) proposed changes to the bTB eradication program; and (vi) benefits of being bTB free.

Two of the researchers, taking handwritten notes, were present at each interview. After the interview, notes from both researchers were compared in order to transcribe the main arguments expressed. The review of the transcription of the different exploratory interviews was done in different steps. In a first step, the transcription of the exploratory interviews was send to all the team members (paper’s authors) and then we organised a group meeting where all team members discussed together the results from those interviews. After that, the team of sociologist prepared a first draft of the interview guide for the qualitative in-depth interviews and they send it to all the authors of this paper for the final discussion and agreement.

Interviews in Andalusia were conducted at the beginning of December 2014 (from 1/12 to 11/12), whereas in Catalonia they were performed in two rounds: middle July 2015 (from 17/07 to 22/07) and middle September 2015 (from 15/09 to 21/09).

#### Qualitative In-Depth Interviews

This study phase was aimed at gaining detailed information on the themes that emerged from the exploratory interviews in order to understand perceptions of farmers and veterinarians and their interpretation of problems related to the eradication of the disease in Spain.

A “thematic guide” was developed based on previous results and it provided an orienting framework of the different stakeholder groups.

Overall, 14 veterinarians and 25 farmers were interviewed (Table 1), applying a *maximum variation sampling* strategy in order to identify as many different “speeches” as possible (36). By this way, we aim to sample for heterogeneity in order to understand how bTB was perceived by people holding different social positions in the field. With this strategy in mind, we selected a small number of samples maximising the diversity relevant to the research question. Diversity was achieved by segmenting the sample (both of farmers and veterinarians) through two key criteria guaranteeing very different daily experiences: territorial criteria (high/low-prevalence areas) and type of farming (beef, dairy, and bullfighting farmers). By doing so, we obtained a wide spectrum of daily experiences and points of view, enough to “saturate the discursive space” related to the subject, which is what was intended by our qualitative sampling procedure.

**Table 1:** Structure of the sample for the qualitative in-depth interviews.

	Low-prevalence area (Catalonia)	High-prevalence area (Andalusia)	*N*
Farmers (*N* = 25)	Six beef farmers	Eight beef farmers	14
	Four dairy farmers	Three dairy farmers	7
	One bullfighting farmer	Three bullfighting farmers	4

Veterinarians (*N* = 14)	Three veterinarians of the public sector (official veterinarians)	Four veterinarians of the public sector (official veterinarians)	7
	Three private veterinarians	Four private veterinarians	7

Total	17	22	39

Semi-structured face-to-face interviews, lasting between 90 and 150 min, were used for this study phase in order to provide in-depth understanding of the participant’s perspective and, at the same time, to allow all opinions and viewpoints to be brought up during interviews. Only one interviewer was present for each interview (an anthropologist in Andalusia and two different sociologists in Catalonia). Interviews were tape-recorded and transcribed by the team of sociologist and anthropologists.

Prior to the interview, a formal letter (headed by the university logo and signed by the research team) was hand delivered to each interviewee and permission was secured at all levels. Participants were informed about: (a) the purpose of the study; (b) the research team members and their university department (with the address, telephone, and email of the main researcher); (c) the freedom to accept or not to do the interview and to withdraw from it at any time; and (d) the explicit guarantee of anonymity and confidentiality of their personal opinions. Interviews only took place after they were read, and verbal consent was obtained from each participant.

In order to make respondents as comfortable as possible during the interview and encourage them to talk extensively and “freely ramble on,” all in-depth interviews started with a few general questions, which respondents could answer easily. These questions were related to their professional career, type of livestock farm, daily working activities (i.e., activities performed in current job position, in the field, in the farms, etc.), and variation in their workday across the year. As the interview progressed, the interviewer gradually introduced new elements in the conversation directing it to more specific and targeted topics.

Interviews in Andalusia were conducted and transcribed between March and October 2015, whereas in Catalonia they were conducted and transcribed between January and June 2016.

To ensure the protection of sensitive data, recordings and transcripts were stored by the research team, and access to them is reserved exclusively for members linked to this research, who have undertaken to maintain the confidentiality and anonymity specified in the mentioned letter. All the real names of individuals and companies, entities, or institutions were eliminated in order to ensure anonymity. Instead, an alphanumeric code that identifies each sample was assigned to each interviewed person. Each interviewee was warned that if any of the phrases pronounced during the interview were used to illustrate results in some public document, and that in no case would the person’s name be mentioned, but replaced by the mentioned code or attributed to the sample as a whole.

An ethnographic methodology was used in this study. Interview transcriptions were analysed through a method inspired on the grounded theory approach, based on the constant comparisons between data of the whole dataset (of all transcripts) and on the use of a repeated coding, which provided a scheme of the main perceptions, opinions and beliefs circulating in the discourses of the study population (37). The records of the interviews were examined thematically by noting and coding each piece of information in the transcriptions. The coding allowed highlighting all central emerging themes. In relation to the internal reliability, the interviews’ transcriptions were compared and discussed between three different members of the research team. Each researcher did it separately, and they met to agree on the relevance of the emerging themes and its interpretation. A single meeting was enough to agree on a common interpretation because there were no major discrepancies.

For each theme that emerged, the most representative sentences were transcribed in their original language (i.e., Spanish or Catalan) and included in the Supplementary Material. From here onwards in the text, we will refer to each sentence as {S*n*}, where “S” means “sentence” and the “*n*” is an integer number whose value represents the unique identifier of the sentence.

## Results

### Exploratory Interviews

Following the general script previously described, the exploratory interviews allowed us to identify the following themes to be further investigated in the second study phase.

#### Strong Points of the bTB Eradication Program

**Figure 2:**
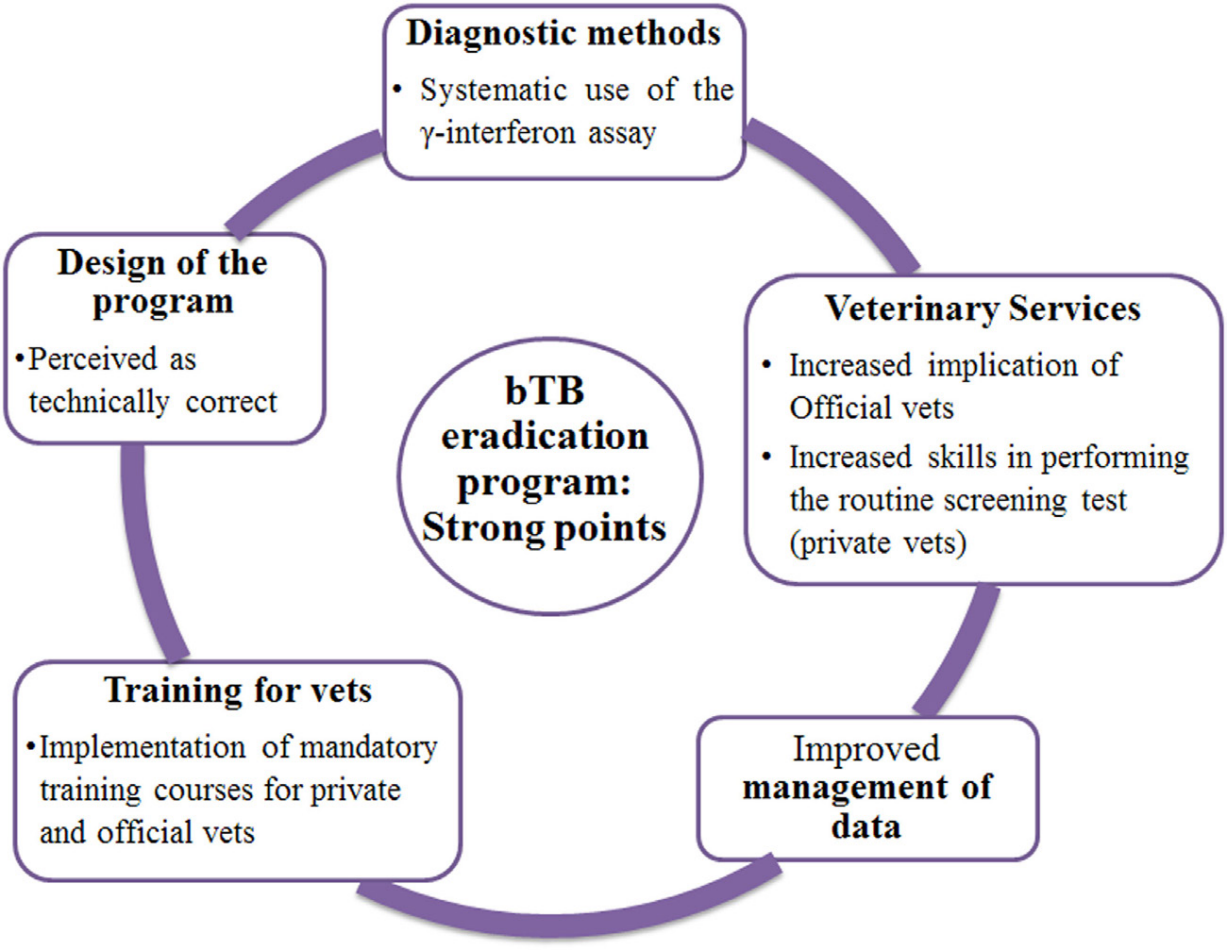
Schematic representations of the main themes emerged from exploratory interviews as “Strong points of the bTB eradication program”; results for Andalusia and Catalonia are presented together. “vets” = veterinarians.

In general, the programme was perceived as technically correct. The increased implication of veterinary services, the systematic use of the interferon-γ assay (IFN-γ), and the implementation of mandatory training courses for veterinarians (public and private) organised by the Spanish Ministry of Agriculture and Fisheries, Food and Environment were perceived as major improvements of the programme in the last years (Figure 2).

#### Weak Points of the bTB Eradication Program

**Figure 3:**
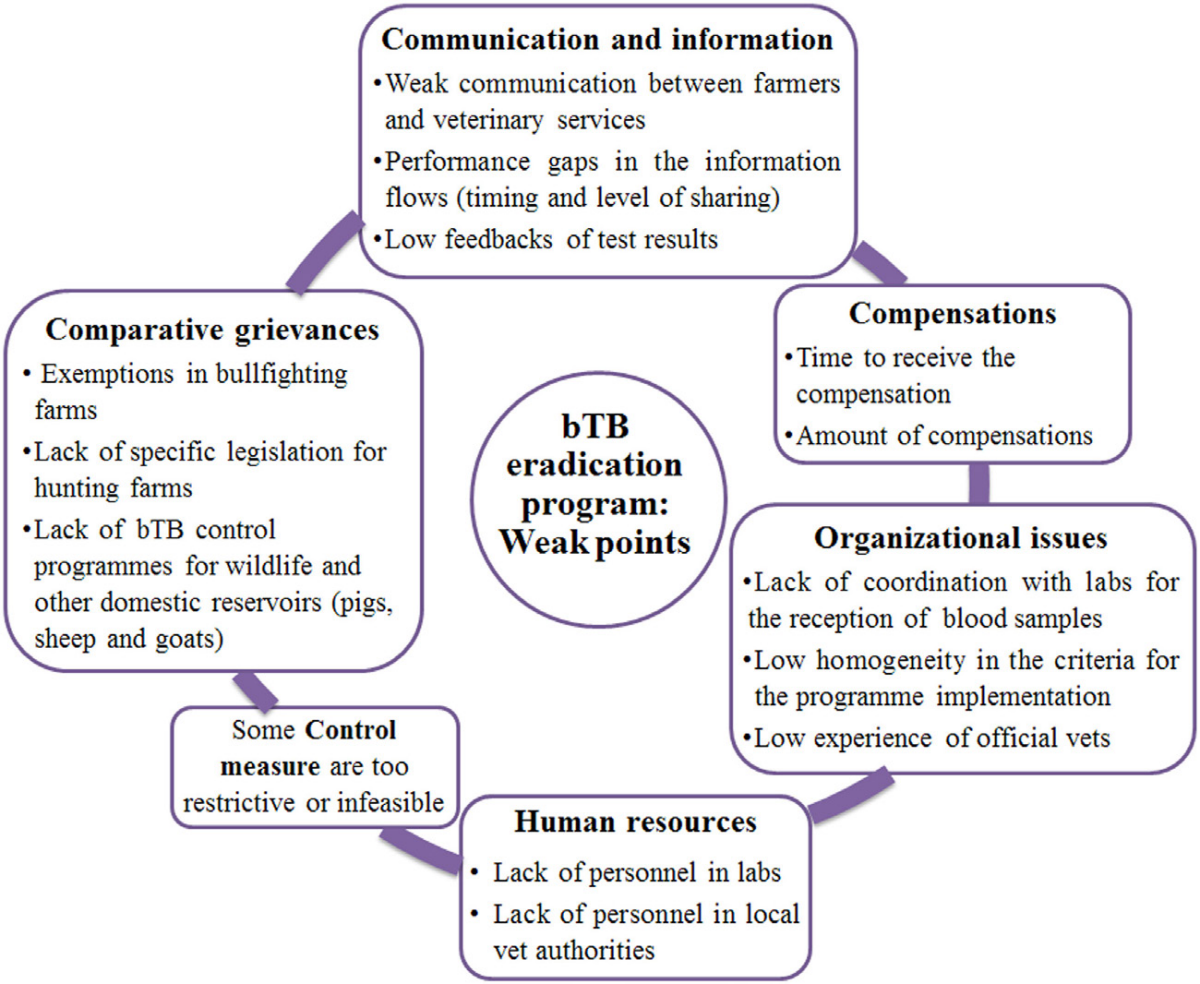
Schematic representations of the main themes emerged from exploratory interviews as “Weak points of the bTB eradication program”; results for Andalusia and Catalonia are presented together. “vets” = veterinarians; “labs” = diagnostic laboratories.

Main weak points were related to the communication flow, organisational issues and the suitability of the human and economic resources currently assigned to the programme (Figure 3).

Concerns were expressed in relation to the coordination with the labs, the experience of official veterinarians who supervise private veterinarians in performing the single intradermal test (SIT), the lack of homogeneity in the implementation criteria of the bTB eradication programme and the lack of human resources. Interviewees also mentioned that some of the implemented control measures were too restrictive or infeasible.

Some stakeholders reported the comparative grievance that is generated due to the special legislation that is in place for bullfighting herds, as in herds with cattle that is older than 24 months bTB testing is not performed. Moreover, the presence of wildlife and other domestic bTB reservoirs not included in the eradication programme was perceived as a comparative grievance by farmers and contributed to generate uncertainty on the achievement of bTB eradication.

#### Reasons for the Failure of bTB Eradication

**Figure 4:**
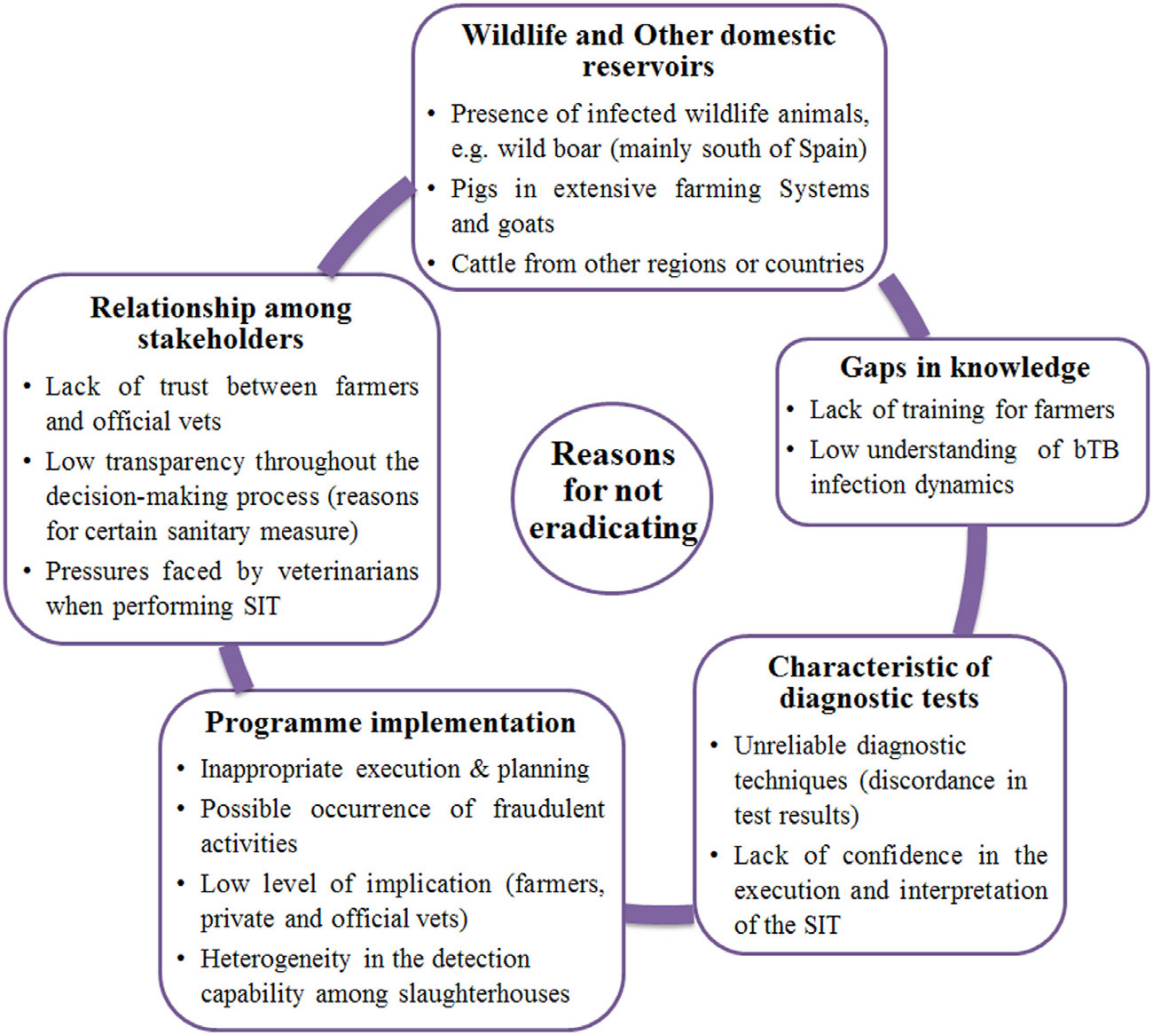
Schematic representations of the main themes emerged from exploratory interviews as “Reasons for the failure of bTB eradication”; results for Andalusia and Catalonia are presented together. “vets” = veterinarians; “SIT” = Single Intradermal Test.

Arguments that emerged in this section were related to the lack of confidence in the results of the diagnostic tests, the heterogeneity in the bTB detection capacity among the different slaughterhouses, the relationships among stakeholders and pressures faced by private veterinarians when interpreting the skin test (Figure 4).

The importance of the level of implication of the different actors in the bTB eradication programme (i.e., farmers, private, and official veterinarians) and the lack of trust between farmers and official veterinarians were also mentioned.

Moreover, the reason for certain sanitary measures was somewhat unclear or not well understood and the presence of infected wildlife animals was perceived as a major obstacle for the bTB eradication, especially in the south of Spain.

#### Future Perspectives

**Figure 5:**
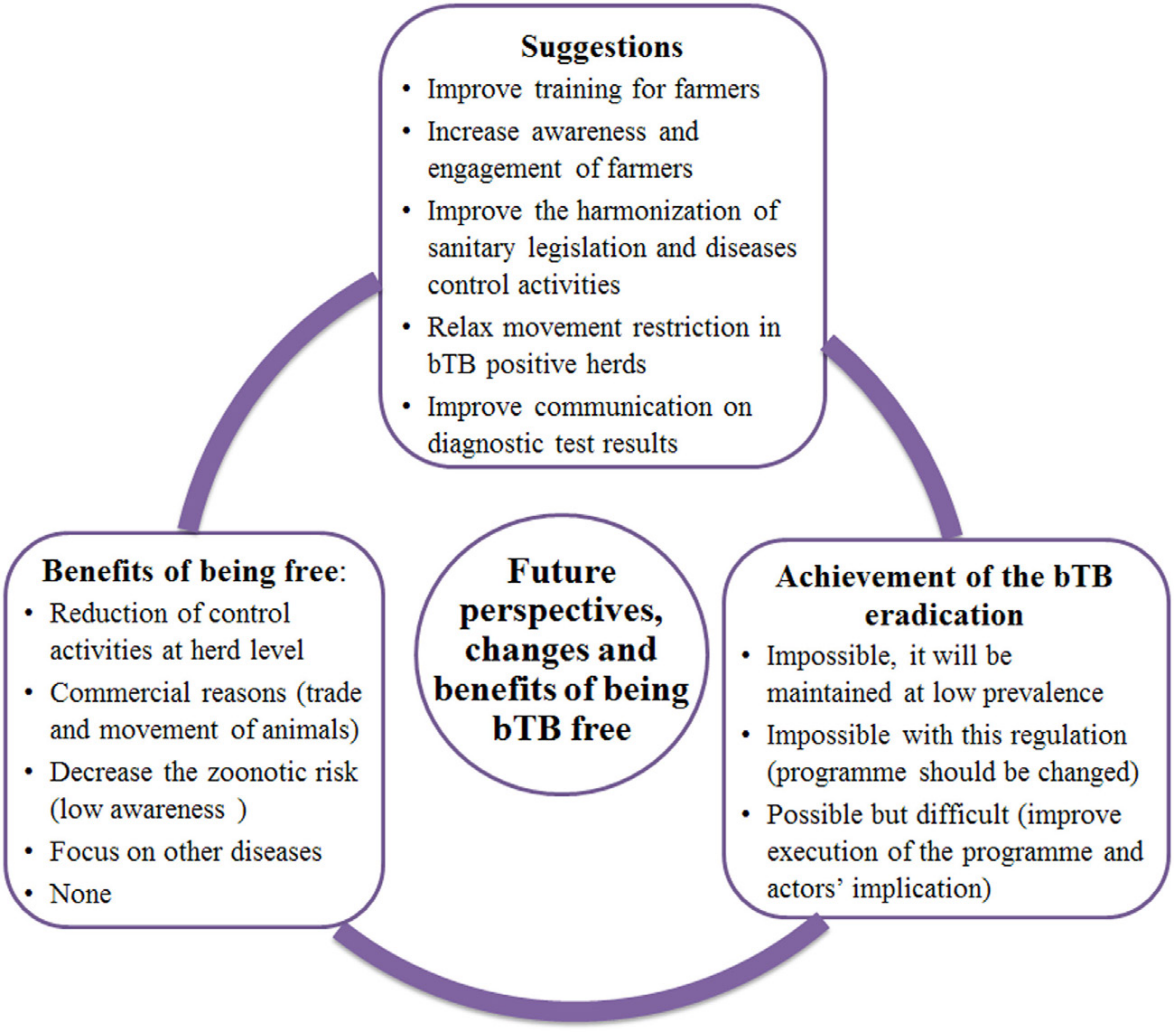
Schematic representations of the main themes emerged from exploratory interviews as “Future perspectives,” “Proposed changes to the bovine tuberculosis (bTB) eradication program,” and “Benefits of being bTB free”; results for Andalusia and Catalonia are presented together.

In this section, very different views were expressed (Figure 5): some people considered that it was at all possible to eradicate the disease and others considered that it will only be possible to maintain a low prevalence.

#### Proposed Changes to the bTB Eradication Program

The different stakeholders considered that improvements to the bTB programme should focus on training (especially for farmers) and communication. It was also mentioned that measures related to movement restrictions should be relaxed (Figure 5).

#### Benefits of Being Free of bTB

With the exception of some awareness on the potential zoonotic risk of bTB reported from some people, few benefits of being bTB free were perceived (Figure 5). The perceived economic impact of the disease was mainly related to the consequences of animal movement restrictions and, therefore, benefits of being bTB free were mainly related to the reduction of control activities at herd level (i.e., frequencies of routine screening) and the removal of restrictive measures on animal trade.

Based on these results, we developed a thematic guide to be used in the qualitative in-depth interviews (Table 2) which outlined the most relevant themes identified and itemised as follows:

(i) bTB detection and control (reliability of diagnostic techniques, organisation and human resources, measures provided for by the programme).
(ii) Training, information, and communication (training for farmers and veterinarians, level of implication of different actors, and communication and information flows within and between levels and categories).
(iii) Role of wildlife and other domestic reservoirs (wildlife reservoir and other domestic species, game hunting areas and farms, specific legislation for bullfighting farms).
(iv) Perception of social aspects (i.e., reciprocal relationships among stakeholders).
(v) Risk perception on bTB and benefits of eradication (risk perception of economic aspects, such as costs of implementing the programme or direct and indirect losses due to the disease).
(vi) Future perspective on the progress of bTB and proposed changes to the programme.

**Table 2:** Thematic guide (topics and example questions) used in the qualitative semi-structured in-depth interviews aimed at gaining detailed information on perceptions and opinions of farmers and veterinarians about the bovine tuberculosis (bTB) eradication programme in Spain.

❖ *Establishing the first contact: Short opening questions* ➢ What is your professional career path? ➢ What are your main daily work activities? (i.e., activities performed in current job position, in the field, in the farms, etc.) ➢ What is your typical workday like? How does it change throughout the year? ➢ What is your experience with the eradication programme? (if not already mentioned)
❖ *Topic 1: Evaluation of the bTB eradication program and control measures (adequate, insufficient, excessive, or illogical)* ➢ Are frequencies of routine bTB screening adequate? ➢ Ask about diagnostic test: reliability of single intradermal test (SIT) and the interferon-γ assay (IFN-γ), differential diagnosis and diagnostic interference with PTB. ➢ Coordination with labs and availability of diagnostic kits for the interferon-γ assay (IFN-γ). ➢ SIT execution: are good practice applied? (i.e., cutimeter use, measure fold, etc.) ➢ What do you think about the official controls on the execution of the SIT? (adequate, insufficient, excessive…). Should they be addressed appropriately? How? ➢ What do you think about the sector-specific legislation for bullfighting herds? (bTB screening exemption for cattle older than 24 months, legal argument that justifies this measure). ➢ Do you think that the applied control measures are adequate? Too strict? Are they feasible and applicable? (existence of fraudulent activities, reasons for fraudulent activities to occur, effects of administrative pressures on fraud, and motivation)
❖ *Topic 2: Other reservoirs* ➢ What do you think about the role played by wildlife species in the maintenance of the disease? Is it a real problem or just an excuse? Is the administration doing enough to control and solve this matter? ➢ What do you think about hunting areas and activities, hunting farms and the mixed hunting-farming subsistence strategy? ➢ What do you think about the role of other domestic species? (sheep, goats and pigs in extensive systems, others…)
❖ *Topic 3: Perception on social aspects, management, and organisational dynamics* ➢ Relationship with other social factors and institutions (dependence, confidence, mistrust, and mutual perception): Official and private veterinarians. Private Vetrinarian group (ADGS). Slaughterhouses (evaluation of activities). Farmers and farmers’ association. Veterinary medicine companies. Administration (evaluation of communication and administration operations). ➢ What you think about the organisation and the mode of operation of the ADGS? ➢ Inter- and intra-institutional coordination (between different Ministries or between central and local level of the same institution). ➢ Implication and transparency of administration (particularly in respect to the diagnostic test results). ➢ Information and training for farmers and veterinarians (level of dissemination, evaluation of courses and events on bTB, etc.). ➢ What kind of information, format, and method would be the most effective and appropriate to train the different groups about the risk of bTB and its control?
❖ *Topic 4: Risk perception on bTB and its economic impact* ➢ Do you think bTB can produce direct and indirect losses on production? ➢ Do you think bTB can represent a risk for human health? ➢ Are the human resources destined to the implementation of the bTB eradication programme adequate? (impact on testing frequencies and test execution) ➢ What do you think about the administrative sanctions and their application? Are they adequate? ➢ What do you think about the farm subsidies? Are they adequate? May they influence farmers’ decision process regarding management of animals and farm’s infrastructures? How? ➢ What do you think about financial compensation paid to farmers for the slaughter of bTB test-positive cattle? (adequacy of compensation, agility of procedures, etc.) ➢ Influence of the farming type and farms’ characteristics to the correct implementation of the programme (i.e., difficulties due to the extensive farming system, adequate state of, reluctance among bullfighting farmers to test animals for difficulties in management).
❖ *Topic 5: Proposed changes to the programme* ➢ What would you change of the bTB eradication programme? ➢ Would you improve some control measures already in place?
❖ *Topic 6: Future perspective on the progress of bTB* ➢ What are main benefits to be bTB free? ➢ What do you think on the failure of bTB eradication campaign? ➢ Is the failure of bTB eradication mainly due to the persistence or to a continuous spread of the disease? ➢ Can the eradication be achieved? How? When?

### In-Depth Qualitative Interviews

Main results obtained from the ethnographic reports of both areas are described below. Since we did not observe major differences in attitude and opinions between the two study areas, results are presented together, highlighting differences when these were identified.

#### bTB Detection and Control

A generalised lack of confidence in the bTB diagnostic tests clearly emerged during the in-depth interviews. Both farmers and veterinarians expressed strong uncertainties on the reliability of test results, although this perception was widespread especially among farmers; so much that some people used the term “lottery” when explaining their perception about test results {S1}. Actually, farmers expressed that they do not want to have any bTB-infected animal in their herd, but that they want to be sure that the test-positive animal is truly infected {S2}.

Uncertainties were mostly associated with the SIT and mainly attributed to the lack of confirmation of positive results and they asked for the application of complementary tests for the verification of the final results {S3}. Reasons provided were the absence of visible lesions in slaughtered animals {S4}, discordance of results between the SIT and the IFN-γ {S5} and the use, as screening test, of the SIT instead of the single intradermal comparative cervical test (SICCT), as it could give cross-reactions with paratuberculosis or other environmental mycobacteria {S6}.

Concerns with the existence of false-negative results were also mentioned but mainly by the official veterinarians and related to bad practices in the field and erroneous execution of the SIT. This group, more than others, disagreed on the systematic use of the SICCT and defended the use of SIT as the screening test. Even though, they admitted a certain degree of subjectivity in the interpretation of the SIT results and a great influence of the level of experience of the veterinarian in question {S7} emphasising and warning about the importance of the professional training of veterinarians {S8}.

Private veterinarians also highlighted that a correct application of the SIT is not always easy as some cattle are difficult to manage and farms do not always have the necessary infrastructure. The importance of having good infrastructure was highlighted by several interviewed, not only to correctly perform the SIT but also to prevent veterinarians from risk of injuries and lesions. The lack of support from the official veterinary services to ensure the existence of adequate infrastructures for bTB testing {S8b} was also mentioned.

On the other hand, the IFN-γ was generally perceived as a better diagnostic test than the SIT; thus, its introduction and systematic use was perceived as positive by most of the participants in the study {S9}. Especially, veterinarians highlighted that the IFN-γ is a valid and helpful tool to dispel doubts on diagnostic results {S10} and that it reduces pressure on veterinarians during field activities as it is performed in labs {S11}. However, some concerns were expressed on the IFN-γ regarding the possible existence of false-positive animals {S12} and the high cost of this diagnostic test that makes its systematic use not always feasible {S13, S14}. Furthermore, the difficulties in sending blood samples to the laboratory on time from remote areas and the lack of support from the labs {S15} were also reported. Finally, another issue mainly expressed by private veterinarians and farmers was the over-saturation of some laboratories and the consecutive delay in receiving the results due to the lack of coordination {S16}; on their side, official veterinarians acknowledged that organisational problems have happened in some occasions due to the lack of enough personnel in the lab. Lack of enough human resources for bTB activities was also related to a deficient post-mortem inspection in the slaughterhouses or field activities supervision {S17, S18}.

Another important issue that emerged in relation to the perception of the diagnostic techniques as unreliable was the lack of understanding of test results (e.g., doubtful results in animals around 1 year of age). Both farmers and private veterinarians mentioned experiences with doubtful results that nobody has been able to explain and clarify {S19, S20}, and they asked for further investigation and more efficient dissemination of information {S21}.

In the last few years, official veterinarians were in charge of supervising the performance of the skin test done by the private veterinarians. This has generated some conflicts as some private veterinarians consider that the official veterinarians who have to supervise them do not always have sufficient experience {S22}. Furthermore, the eradication programme in areas of high prevalence (as is the case of the south of Spain) has established a stricter lecture of the SIT in infected farms by which doubtful results are considered as positive. This measure has not been well accepted by the interviewed farmers and private veterinarians who would wish to verify positive results {S23}, whereas official veterinarians do think that it is a good change that will benefit the eradication programme.

The screening intervals set by the bTB eradication programme for routine testing were considered functional and adequate by official veterinarians and most of private veterinarians and farmers, albeit they asked for more coordination among different sanitary controls to avoid generating stress in animals and workers {S24}.

Only in certain rural areas of Andalusia, the implementation of two screening round per year was perceived as excessive, especially by farmers, due to the difficult management of beef cattle in extensive farming systems. In addition, farmers expressed the management difficulties that they face during the bTB testing, especially in those farms with extensive managements or in bullfighting farms {S25}. Direct loses due to abortions, work hours, injured animals, and decrease in milk production were mentioned as a major issue related with bTB testing, especially in those infected herds subjected to a high frequency of tests.

Some criticisms were reported in Andalusia with regard to the sector-specific legislation for bullfighting cattle farms (bTB screening exemption for cattle older than 24 months), although different points of views were expressed {S26–S28}. Some interviewees considered that no exceptions should be allowed with bullfighting animals, while others justified this measure and evaluated it as reasonable on the basis of their difficult management, the risk of injuries in animals of high value or changes in their behaviour making them unfit for bullfighting {S29}. However, even within the group of farmers that agree with the exemption of bTB testing, not everyone agreed with the argument of difficult management as still these animals are subjected to other health measures (such as vaccination or deworming). The high genealogical value of bullfighting animals and the economic difficulties that the sector is going through were considered as more relevant for these persons.

In relation to the control measures provided by the programme, the huge economic consequences derived from movement restrictions was mentioned, especially for those farms without infrastructures for fattening animals. This measure was perceived as too restrictive and as the origin of fraudulent activities. Nevertheless, in the last few years, farmers have been allowed to send these animals to specific fattening units; a measure that has been positively received, despite that calves are sold at a lower price {S30}.

#### Training, Information, and Communication

An improvement in the application of the bTB programme in the last few years was highlighted and mainly attributed to the organisation of mandatory training courses. Both official and private veterinarians acknowledged that some bad practices in the field were largely caused by a lack of knowledge and training among veterinarians {S31}.

Official and private veterinarians also expressed the importance of organising such activities also for farmers, ensuring that they could have access to all the available information by increasing awareness and knowledge on the diseases as well as on its impact to the farm {S32, S33}. Some of the interviewees also emphasised the importance of training for farmers in order to improve the understanding of sanitary measures provided for the bTB eradication programme and increase its acceptability {S34}.

Among farmers, the lack of understanding of test results and control measures gave rise to some disbelief and to different guesses, for example, that a high mutability rate of the Mycobacterium invalidates the diagnostic tests and that bTB is just an excuse to reduce the cattle population in Southern Countries {S35}.

It was not clear which should be a more efficient way to deliver such training as some people expressed concerns due to the high number of courses that are already organised for farmers {S36} and a lack of motivation in relation to animal health by some of them {S37}. Among the different stakeholders, private veterinarians were identified as one of the more adequate professionals to inform farmers and raise their awareness on the disease, as they are the ones that usually inform farmers on other matters {S38}.

In relation to the effectiveness of communication between stakeholders, different opinions were reported. On the one hand, some farmers expressed the lack of meeting places to exchange information and to express doubts and concerns on the disease and its control. As a matter of fact, most times they have learnt about the bTB eradication programme and changes in the regulation by talking to other farmers in the bars {S39, S40}.

On the other hand, some other farmers expressed that the communication through their private Vetrinarian group (ADGS) was good enough and they were informed of any changes through them {S41}. Most of the farmers also reported that they would prefer attending informative days about specific issues rather than formal courses and that it would be preferred to organise these meetings during animals’ markets.

Regarding the communication of bTB test results, differences emerged between the two study areas. In Catalonia, it was described by farmers and veterinarians as adequate {S42, S43}; while in Andalusia a general perception of low feedbacks on test results was reported and both farmers and private veterinarians demanded easier and more flexible procedures to get all needed information on lab results {S44, S45}, results of the post-mortem inspections and the cultures {S46}.

#### Role of Wildlife and Other Domestic Reservoirs

The existence of bTB wildlife reservoirs was mentioned as a major obstacle for bTB eradication in Andalusia and Catalonia, but was especially highlighted in those areas with high prevalence and extensive herd management in Andalusia. Different opinions on the role of wildlife reservoirs arose; some people attributed a secondary role in the maintenance of the disease to these species while others were of the opinion that wildlife reservoirs could represent a primary source of infection for cattle {S47–S48}.

In general, controlling bTB in these animals was perceived as a very difficult task and several people expressed the hope of having a vaccine in the future to control the disease in these animals. The development of biosecurity plans to reduce the risk of transmission from wildlife to cattle was also mentioned. However, different views were expressed and some people considered it possible, whereas others considered it impossible to prevent cattle and wildlife interaction {S49}.

Other factors that in the opinion of some people increased the risk of bTB transmission was related to hunting activities and the lack of biosecurity, as different groups of dogs, vehicles, people, etc., interacted with infected wild animals and could spread the disease to other places {S50}. In this regard, farmers and veterinarians agreed on asking for more controls in wildlife, especially in hunting farms as they are managed as livestock farms {S51–S52}.

Several interviewees negatively perceived the supplementary feeding for hunting purposes, as it was linked to an increase of wildlife population and as a consequence an increased risk of infection for cattle herds. Moreover, the economic benefits provided by hunting activities was suggested to lead to the establishment of several mixed farms (wildlife and cattle), therefore, increasing the risk of bTB transmission. In this sense, the importance of the coordination between the different governmental statements responsible to manage animal health and the environment was highlighted {S53}.

In relation to other bTB domestic reservoirs not subjected to any control programme, the potential role of goats, sheep, and extensively reared pigs (the latter particularly in Andalusia) was mentioned. The interviewees reported that sharing pasture by cattle and these other domestic reservoirs poses another risk of infection for cattle and complained about the lack of specific legislation for this matter.

#### Perception on Social Aspects

Although the relationship between farmers was considered to be good, bTB was described as a sensitive issue that is normally avoided in their talks. In some occasions, conflicts between neighbouring farmers were generated to the perception that the adjacent farm was responsible for the bTB infection of the herd as the neighbouring farmer has not complied with the eradication programme and has been the source of the outbreak {S54, S55}.

The relationship between farmers and private veterinarians was described as good as in general, it is an enduring relationship and farmers tend to have a very high confidence on them {S56}. However, the existence of a “patronage relationship” between some farmers and private veterinarians was also mentioned, because private veterinarians conduct in the farm other duties than only the bTB testing that are paid by farmers. This fact could generate pressure on private veterinarians, which might not always act with professionalism as could be strongly influenced by the consequences for farmers due to the bTB control measures and for the fear of losing “customers” {S57}. In this regard, some of the interviewees also mentioned that sometimes the pressure faced by veterinarians generated conflicts, as the most rigorous veterinarians were not well accepted by all farmers {S58, S59}. In this sense, to have a greater support from the official veterinary services was perceived as a way to reduce pressure to private veterinarians {S60}.

There were different opinions about the relationship between official veterinarians from LVSs and private veterinarians and farmers. Some people reported to have a close and effective relationships and a good coordination with them, despite official veterinarians have the role to control and inspect them {S61, S62}. Others described the relationship as tense and of mutual mistrust. Main reason for this difficult relation was due to the perception of fraudulent activities with bTB testing.

The existence of fraudulent practices was acknowledged by some farmers, however, they also argued that, even though not all farmers act the same, they are all treated the same way, and they perceive that the official veterinary services are treating all of them as “delinquents” {S63, S64}.

Concerning the fraudulent practices, the missed communication of animals with doubtful test results and the non-rigorous reading of the SIT were the most reported by both farmers and veterinarians {S65, S66}. These behaviours contributed to generate demotivation especially among farmers but also among veterinarians {S67, S68}.

#### Risk Perception on bTB and Benefits of Eradication

Some differences arose between groups on the perceived burden of the bTB. Official and private veterinarians acknowledged both the health and the economic impact of the disease. They emphasised that animal health is the base of the development of the livestock sector and it is fundamental to an efficient animal production and, therefore, to food security and human health {S69}. The group of veterinarians expressed the need to eradicate bTB also because it represents a public health problem, not only because of the obvious trade benefits but also because of the positive repercussions on animal health {S70}.

On farmer’s point of view, bTB is not seen as an important animal health problem. Most of the farmers perceived that benefits of eradication were mainly commercial, as bTB was not considered having an impact on public health neither a disease causing production losses. The fact that the meat from infected animals can be passed as “fit for human consumption” after the removal of the affected tissue (unless the carcass is generally emaciated and the lesions are generalised) generated doubts about the public health implications of bTB {S71–S73}. Moreover, they strongly disagreed that veterinary services focus so much on bTB instead of controlling other diseases that they consider more severe for human health {S74}.

Generally, farmers did not perceive any production losses due directly to bTB and some of them referred that bTB does not affect animal at all. Only few farmers perceived a direct relationship in the long term between the productivity of animals and the presence of the disease {S75, S76}. In this sense, veterinarians admitted that due to the early detection of the disease, most infected animals do not develop lesions and, in this context, it is difficult to make farmers aware on the impact of the disease {S77}. Thus, farmers mainly perceived the control of bTB as an imposition rather than a necessary activity to protect their animals {S78, S79}. They also mentioned that few studies have been done so far to quantify production losses due to bTB in the current epidemiological context and asked for updated scientific evidence on it. Nevertheless, the economic impact of the disease was strongly underlined by all interviewed groups and the commercial consequences of being bTB positive were perceived as worrisome {S80}. It was reported that some farmer abandoned the sector due to economic cost faced for the control of bTB. This is because, despite the fact that the central veterinary service provides the diagnostic tests and current law provides for indemnity for slaughtered cattle, farmers assume the rest of the costs, mainly due to restrictions on trade and animal movements and field activities for the routine screening (i.e., Vetrinarian for screening, extra-personnel for animal management, derived damages on animals) {S81}.

With regard to the amount of the indemnification, veterinarians generally opined that it is adequate and that increasing indemnity payments would mean rewarding the maintenance of the disease; they also reported that no significant complaints have been received from farmers {S82, S83}.

#### Future Perspective and Proposed Changes to the Programme

Most of the interviewees were sceptics on the possibility of eradication mainly due to the presence of wildlife and other domestic reservoirs. The possibility of maintaining the disease at low levels was seen as the more realistic option but it was conditioned to the existence of a stable regulation {S84}.

Some farmers also doubted about the need of so restrictive measures (slaughter of positive animals, movement restrictions, etc.) taking into account the possibility of developing a vaccine for cattle {S85}. Others would prefer to live together with the disease rather than applying such restrictive measures that, on their opinion, will end up penalising the cattle industry in the country {S86}.

Suggestions and changes proposed to the programme were related to the main problems highlighted, for example, more investigation on diagnostic test, to improve the control on fraudulent activities, to increase the personnel of the LVSs and the implementation of controls plan also on other reservoirs and wildlife.

## Discussion

The continuous evaluation of the bTB programme, in order to identify limitations and modifications needed, requires taking into account the “*non-biological*” context, as it might influence the effectiveness of the eradication plan (16). However, despite the acknowledged importance of these “non-biological” factors, few studies have attempted to evaluate them (38–41) and they have mainly used structured questionnaires.

In this study, we used a qualitative approach in order to identify social aspects that may influence the effectiveness of the Spanish bTB eradication programme. The use of qualitative methods, such as the semi-structured interviews that we used in this study, might have some advantages in relation to the use of structured questionnaires for these types of studies. The main advantage is the fact that they allowed to develop long conversations through which people could describe their personal experiences and opinions in their own words. This generates a discourse that is neither fragmented nor pre-coded, as it happens with structured questionnaires (42). However, it is worth taking into account that qualitative interviews (as well as surveys) can inform on what people say they do, but not what they actually do. These means that the objectively knowledge about their daily practices and perceptions would require the use of other techniques, such as participant observation or systematic observation methods (43). In order to reduce this bias, in-depth interviews were conducted always in private and started with general “warm-up” questions. In this way, we intended to generate an atmosphere of conversation rather than of interview, maximising, therefore, the possibility of achieving honest answers.

A disadvantage of qualitative interviews is that they do not allow making a direct inference of results to the whole population as the number of samples is normally low and the type of sampling is not random. However, this was not the objective of this study as we intended to know the main arguments that are circulating in the study population. In this context, the use of purposive sampling can ensure representativeness and diversity in the obtained results since it allows incorporating people of all possible typologies relevant to the research. This kind of sampling is the most effective technique when one needs to study a certain cultural domain or to explore all existing opinions circulating in the study-populations (44).

Considering both study phases, the main stakeholders involved in the Spanish bTB eradication programme were included in our study. We interviewed cattle farmers (beef, dairy, and bullfighting); Researchers with experience on bTB; Veterinarians working in the diagnostic labs: with responsibilities in the performance of the tests (gamma interferon, culture, etc.) that are performed in the bTB eradication programme; Private veterinarians who conduct bTB testing; and Official veterinarians working at different levels:

(i) Autonomous community level (regional veterinary authority) with responsibilities in the coordination of the programme in their autonomous community. These veterinarians, together with official veterinarians of other autonomous communities, also participate in the technical meetings organised at national level to review and discuss the bTB programme;
(ii) County level: with responsibilities in the coordination of the programme in their area.

Although it is true that some stakeholder profiles are missing, for example, we did not included veterinarians working in the slaughterhouses, trading partners, or consumers; however, we have included representatives from the groups most involved in the implementation of the National bTB eradication programme. Therefore, we believe that the results of this study may have a wide applicability as we have gained information on the main discourses.

Overall, 52 people were interviewed (13 people for exploratory and 39 for in-depth interviews), among those there were 22 veterinarians and 30 farmers. The selected number of participants relied on previous studies based on grounded theory and wanted to maintain a balanced emphasis between the homogeneity (requiring smaller size) and the heterogeneity (requiring larger size) of the sampling target (45, 46). In the case of farmers’ selection, the size of herds, the production type, and bTB prevalence at county level were taken into account; while, in the case of veterinarians, the years of experience working with the bTB programme, their roles and responsibilities at the workplace and the disease prevalence at county level were considered. Doing this, we wanted to avoid failures in capturing insights, experiences, and activities and, therefore, achieve the theoretical saturation of data (45).

In recent years, the application of ethnographic methods has been extended to the description and analysis of social relations within any group of people: social, professional, or conceptual (47), making this strategy of analysis particularly suitable for our study. Moreover, this methodology is optimal if people to interview tend to disguise their way of acting and/or thinking, as could be the case in the bTB eradication programme.

One of the main results of this study was an apparent lack of motivation of some stakeholders and a general feeling of distrust in control measures and disbelief in test results. The complexity of the disease combined with gaps in knowledge and the lack of an efficient communication about the interpretation of diagnostic test results and control interventions seems to be important causes of disbeliefs, which in turn might generate different kinds of guesses and interpretations. Good communication and coordination between the different stakeholders have been previously described as having paramount importance in any health programme, since it might be a critical factor for the success of bTB control interventions (39, 40). The implementation of official communication plans on bTB and the selection of the most appropriate strategy would be an interesting research topic to tackle. Moreover, our results also points out the importance of informal places for discussion and solving doubts and the primary role of private veterinarians influencing farmers’ opinions.

Similar to our findings, Calba et al. (39), in a study conducted in Belgium, reported the key role that private veterinarians have in the surveillance and communication with farmer; they found that private veterinarians are under pressure of their client (farmer), making necessary a greater support by the official veterinary services, and highlighted the importance to address such issues in order to improve the acceptability level of the bTB surveillance system. In agreement with Calba et al. (39), we found that the lack of support by the official veterinary services has mostly likely contributed to the feeling of distrust towards official veterinarians, to the absence of adequate infrastructures to perform the SIT, and to the pressure faced by private veterinarians.

Perceived inaccuracies in bTB detection increased mistrust and demotivation, especially among farmers. Discordant results between diagnostic tests, the lack of guides and standards for interpretation of diagnostic results and the absence of lesions at the *post-mortem* inspection have been already described as possible barriers toward bTB eradication in previous studies, as they might reduce the engagement of farmers in preventive health interventions (4, 8, 39). Our results further highlight that the level of confidence on the interpretation of SIT results was often linked with skills and experience of official and private veterinarians involved in the field activities of the testing campaign.

Along these lines, since expert estimations of the risk of bTB contain many and high levels of uncertainty, it is perfectly rational for farmers not to limit themselves merely to these estimations when evaluating the magnitudes of risks, as stated by some scholars (48, 49). It is, therefore, logical to also ask about such issues as how much trust the institutions involved in risk management deserve: “I have argued that public perceptions of and responses to risks are rationally based on judgements of the behaviour and trustworthiness of expert institutions, namely those that are supposed to control the risky processes involved” (49). The results of our research seem to fit well with this hypothesis, as far as public and private institutions in charge of tuberculosis control are implementing actions perceived as ambiguous or not always coherent by the farmers.

The lack of the application of sanitary measures to wildlife, goats and pigs in extensive farming systems were pointed out and it was perceived as a comparative grievance to what is done in cattle, as measures on cattle were perceived as much more strict. In this regard, all groups asked for improvement in coordination between institutions and implementation of specific measures and better management of wildlife, especially for hunting farms. In this regard, it is worthy to mention that recently it has been launched a reinforced surveillance programme for bTB in wildlife named PATUBES (34) which was not known by the interviewers as it was not publically available at that time. Thus, it would be worthy to update opinions and beliefs in the future in the light of the results of this reinforced programme.

In relation to other domestic reservoirs, the Spanish bTB eradication programme only includes the testing in goats that are epidemiologically related to infected cattle herds, and sheep and extensive pigs are not included in the programme. With the exception of goats (33), the role of sheep and pigs in bTB epidemiology is still controversial, but some stakeholders had the perception that they are important reservoirs. In this sense, more research might be needed in order to communicate effectively their role to the different stakeholders.

Some other factors also mentioned in this study such as some non-specific SIT reactions in young animals might also need further research in order to fill gaps and enhance communication.

Moreover, farmers perceive very few benefits of being bTB free and that the economic impact of the disease is due to its control rather that to its presence. In addition, a low awareness on the zoonotic risk of bTB also emerged; these aspects might discourage farmers in implementing preventive measures against bTB since the cost for such implementation would outweigh perceived benefits. This perception might be another major factor influencing the effectiveness of the programme as preventive measures might be undertaken by farmers if they clearly perceive that the benefits outweigh the costs (4).

The lack of enough human resources for bTB activities, as reported by the group of official veterinary services, might also deserve further attention. The support of official veterinary services to private veterinarians beyond official control inspections could help to enhance relationships and communications between groups.

## Conclusion

The use of a qualitative approach, allowed us to catch specific information related to the local context and highlight aspects that could be missed by applying quantitative epidemiological methods. Our findings represent a good part of the probable sphere of perceptions, opinions, behaviour, attitudes, and knowledge of the study population and several key critical points that may hinder the success of the bTB eradication programme in Spain were identified.

Major issues were related to the perception of the bTB programme as a law enforcement duty and to the lack of an adequate motivation, as a general feeling of distrust towards official veterinary services was expressed. The improvement of communication strategies should be considered as a priority, as it seems to be a major factor influencing the trust between stakeholders and the effectiveness of the eradication plan. Lack of understanding of test results and control measures, lack of perceived benefits of being bTB free, gaps on knowledge together with the complex epidemiology of bTB deserves further efforts on communication. Private veterinarians had a major role in influencing farmers’ opinions but their feeling of inadequate support from veterinary services should be taken into account.

These results can be extremely useful to develop some context-dependent recommendations and interventions in order to increase the acceptability of the bTB eradication programme and ensure its proper implementation.

## Ethics Statement

Ethical approval was not required for this study, in accordance with the national and institutional guidelines, as it did not include samples or experiments on people but only their expression of opinions in relation to a specific topic. With regard to the informed consent of participants: as the interviews were anonymous, the data were analysed anonymously and the decision to participate in the study was solely up to each contacted person, we did not consider it necessary to obtain a written consent. We orally informed all participants of the elements of consent and permission was obtained verbally before starting the interview. At the beginning of each interview: interviewers introduced themselves and the contacted person was informed on the study design and its objectives. It was explained that the participation was voluntary and completely anonymous (data collection and analysis) and that they could stop the interview at any time. It was explained that there were no expected risks and no expected personal benefits associated with participation in the study. We also asked their approval for using information collected through the interview and for using direct quotes from them and these would only be cited as from a “farmer” or “veterinarians,” keeping the anonymity.

## Author Contributions

Conceived and designed the study: GC, AA, and JE. Performed and transcribed qualitative exploratory interviews: GC, AA, SN, and JC. Performed and transcribed qualitative in-depth interviews: PI, EC, and SL. Analysed collected data: GC, AA, PI, EC, and SL. Wrote the paper: GC and AA. Revised the paper: AA and JE.

## Conflict of Interest Statement

The authors declare that the research was conducted in the absence of any commercial or financial relationships that could be construed as a potential conflict of interest.
