# Supplementary material for: Farmer and Veterinarian Attitudes towards the Bovine Tuberculosis Eradication Programme in Spain: What Is Going on in the Field?
Source: Front Vet Sci. 2017 Nov 27;4:202. doi: 10.3389/fvets.2017.00202 (PMC5712013; doi:10.3389/fvets.2017.00202)
Supplement: Supplementary file 1 [file data_sheet_1.pdf]

Farmer and Veterinarian Attitudes towards the Bovine Tuberculosis Eradication Programme in Spain: What Is Going on in the Field?

Giovanna Ciaravino*[1], Patricia Ibarra[2], Ester Casal[3], Sergi Lopez[3], Josep Espluga[3], Jordi Casal[1,4] Sebastian Napp[4], Alberto Allepuz[1,4]

***Corresponding author:**
Giovanna Ciaravino
E-mail: giovanna.ciaravino@uab.cat

Supplementary material

List of Abbreviation

γ-IFN = Interferon-γ assay

SIT = Single Intradermal Test

bTB = Bovine Tuberculosis

A. Sentences from the qualitative in-depth interviews reported in their original language (i.e., Spanish or Catalan)

i) bTB detection and control

S1-"yo no tengo confianza en las pruebas, para mí es un poco de lotería". (farmer)

S2- "...porque yo animal positivo no lo quiero tener ni de coña, aunque sea la mejor vaca, como el mejor toro, o sea, seguro que no lo quiero tener, porque no es más que un problema, pero quiero tener la certeza de que es positivo" (farmer)

S3-"[...] en otros países se hacían las dos cosas a la vez, el γ-IFN y la SIT, y si coincidían era positiva, bueno pues yo me quedaría más tranquilo si me hicieran algo de eso, aunque me cobraran algo más. [...]" (farmer)

S4-"tuve que matar ochenta y tantos animales, de los que no me decomisaron ni una sola pieza de nada y yo ya estaba cabreado" (farmer)

S5-"No es una prueba fiable, que si daba alguna en tuberculina, alguna no daba en el γ-IFN. Y alguna daba en el γ-IFN y en lo otro no, que era muy raro" (farmer)

S6-"A ver quién me dice a mí que muchas de mis explotaciones son de paratuberculosis y no de bTB. Nosotros no hacemos la comparada [...]" (private veterinarian)

S7-"[...] La SIT bueno, da buenos resultados pero con muchos condicionantes que te pueden afectar [...]" (official veterinarian)

S8-"[...] yo he ido al campo y me he encontrado en las charlas con algunos ganaderos de que faltaba rigor en la prueba. Pero no rigor por no quererlo hacer sino rigor por desconocimiento [...]" (official veterinarian)

S8b-"[...] Te encuentras explotaciones extensivas que tienen unas mangas estupendas y tienen unas instalaciones estupendas donde los veterinarios que trabajan en el campo pues están bien, son seguras, son prácticas, son cómodas y otros sitios que están regular. Eso sí que creo que es un punto en el cual la administración de alguna manera deberíamos meternos más, tanto por la ejecución de esto como por temas de prevención de riesgos laborales. . Más de uno se ha quedado en la manga, que o se ha caído… cosas que no ha pasado nada por ahora pero podrían pasar". (official veterinarian)

S9- “Claro al meter el tema del γ-IFN han aparecido no más sino lo que había […]” (official veterinarian)

S10-“[…] y yo tengo duda con ella, el interferón me saca de dudas, ¿entiendes?” (private veterinarian)

S11- “[…] y el gamma, mejora la SIT en algún caso, lo mejora entre otras cosas en el tema de que el diagnóstico es en laboratorio, por lo tanto la presión en el campo desaparece.” (official veterinarian)

S12- “el interferón también es verdad que saca vacas que no son…Hombre, saca falsos positivos, sí…No sé por qué. Eso los del laboratorio sabrán…” (private veterinarian)

S13- “y con esa (γ-IFN) hay gente que quiere que se la haga pero el problema es ese, no hay perras. Y el laboratorio la mitad de las veces no tiene posibilidad de hacerlas”. (farmer)

S14- “El γ-IFN, yo personalmente le pegaba fuego. Aunque es una herramienta válida pero es cara, al ser cara depende de los presupuestos y una campaña de saneamiento continua no puede depender de que ahora tengo dinero, y mañana sí y mañana no […] Y esto no puede ser. Entonces, y esto ya lo he dicho más de una vez, mejor que el dineral tan grandísimo que cuesta los kits de γ-IFN lo invirtiésemos en más personal […]”” (official veterinarian)

S15- “tantos problemas porque tienes que llevarlo al laboratorio que nosotros, incluso yo le digo a tus compañeros, nos pusieron un límite de entregar la sangre, y aquí hay como poco hasta 100 km hasta laboratorio y no nos esperaban” (private veterinarian)

S16- “cuando todo el mundo saneamos al mismo tiempo, llega mucha sangre al laboratorio y ese laboratorio incluso…, a nosotros nos ha llegado a tardar 28 días en sacar los resultados, de publicarlos o meterlos en la base de datos”. (farmer)

S17-“[…] Mandamos partidas a matadero que no se muestrean, porque hay varios mataderos y no tiene personal para cubrir todos los días que se matan en todos los mataderos.” (official veterinarian)

S18“…vamos a una finca, vamos a cargar animales, y vamos a la finca y nos dicen “No, no están, están allí”. Están en la finca de un vecino porque tiene mejores corrales para cargar que está calificado sanitariamente que es T3, sin embargo el que está cargando allí es positivo y está cargando en los corrales de otro que es negativo. Eso te enteras si vas a la finca, si no, no te enteras” (official veterinarian)

S19- “Hay otra cosa que no me acaban de aclarar y no lo acabo de entender porque yo creo que nadie lo sabe tampoco, a no ser que viniera un inmunólogo y nos lo explicara de puta madre […] deben ser reacciones esas a algún Mycobacterium, algo raro” (private veterinarian)

S20-“ese caso se expuso allí el día de la reunión, y tanto ni veterinarios ni como ganaderos ni técnicos, ninguno sabía darle una explicación.”(farmer)

S21-“[…] tendrían que investigar más y cambiar el sistema para atacar la enfermedad, porque vamos yo para mí está demostrado que se han matado muchos animales y que no se ha conseguido nada, entonces habría que cambiarlo, digo yo” (farmer)

S22- “A nosotros nos está haciendo controles gente menos preparada que nosotros. Es que por eso se quejaban algunos compañeros, que han puesto un recurso de que no le hagan controles, que el que vaya que esté más preparado que él”. (private veterinarian)

S23-“Hombre, si va a calificar y no da ninguna positiva, […], le estás jodiendo la calificación que le hace falta para poder vender. Entonces es absurdo, pues antes las repetíamos”. (private veterinarian)

S24-“Yo lo que pediría de alguna manera es que se coordinara lo que es el saneamiento con los demás controles, para que una vez que tú tengas el ganado recogido y dispuesto a pasar por la manga, se hiciera todo de una vez, que no haya que estar trastornando a los animales

tantas veces que algunas veces a nosotros nos hace falta aquí a la mujer, los niños, mi primo, el otro, la mujer del otro…”. (farmer)

S25-“En cuello se hace, pero en ganado bravo, ponerte a hacerlo en cuello…, muchas veces te estás jugando el propio físico, en meter la mano hay en la mangada para pelarle el cuello, medir, pinchar… Eso es complicado. Porque el cuello es un parte de bastante movilidad animal” (farmer)

S26-“no puedes proteger a unos ganaderos así y a otros no. […] en este tema yo creo que están perjudicándolos más que beneficiándolos. (private veterinarian)

S27-“habría que aplicarles a todo el mundo lo mismo, lo veo así de claro. Todo lo demás yo pienso que es esconder el problema” (official veterinarian).

S28-“[…] no sé, a lo mejor es lógico que…, también si los pasas por una mangada se te puede fastidiar un cuerno, de un animal de esos que valen…” (farmer)

S29-“Hay quien dice que el animal cuanto menos se toque mejor, porque está más en libertad, más salvaje.”(farmer)

S30-“Claro, sí se aprovechan, cuando van a matadero sucio se aprovechan pero bueno hubiera sido un mal menor, pero el problema gordo es tenerles que dar a los animales comida durante un montón de tiempo…” (farmer)

ii) Training, information and communication

S31 - “[…] en la bTB hemos ido saliendo de la universidad y han dicho echarse al campo y nos ha ido enseñando un compañero y como nos ha enseñado el compañero y han ido haciendo la prueba y dando un diagnóstico. […] en el curso me he dado cuenta de que eran conceptos de partida que no era ni porque ellos querían hacerlo peor sino que lo habían aprendido así y no habían visto la reacción de bTB claramente” (official veterinarian)

S32-“[…] Yo ahora mismo estaría por hacer lo mismo que se ha hecho con el tema de los veterinarios a nivel de ganaderos.” (private veterinarian)

S33-“[…] la trasmisión de la información y la implicación de los ganaderos. Yo creo que eso es fundamental, y cosas que estamos ahí flojeando. Oye mira esto es así, asá y explicárselo a todo el mundo” (official veterinarian)

S34- “[…] Es que no tiene mucho sentido que te digan que esto es muy importante y después no te expliquen cómo funciona, ¿entonces cómo podemos combatirlo? ¿No?” (farmer)

S35- “[…] yo mi percepción, igual estoy equivocado, pero mi percepción es que esto se debe a cuestiones políticas, es decir, por intereses políticos europeos, interesa reducir la cabaña bovina en España y están siendo muy duros con esta enfermedad para reducir la cabaña bovina, porque si no pondrían…, si realmente fuera un problema pues investigarían más, pondrían más medios, lo harían de manera igualmente estricta con bovino y con otras especies” (farmer) […] (farmer)

S36- “Que no creo que sea sólo cuestión de cursos, que últimamente hemos visto que venga curso para esto, curso para lo otro, y están los pobres aburridos, que si curso de bienestar animal en el transporte, bienestar animal en la explotación, usos de biocidas en la higiene veterinaria,…” (private veterinarian)

S37- “Es muy difícil porque ellos tienen otras preocupaciones que no es la de la sanidad. La sanidad el ganadero no es consciente realmente de la importancia directa e indirecta que pueda tener [...]”. (private veterinarian)

S38-“Los veterinarios de la ADSG informan a todo el mundo, ya que él quiera o no quiera, eso… Pero informar, informan” (farmer)

S39-"[…] A part del que es puguin explicar als bars no existeixen espais de trobada pels ramaders" (private veterinarian)

S40-"[…] Si hi ha canvis a les lleis o alguna cosa ningú els hi explica. Potser els explica un altre en un bar." (farmer)

S41-"[…] A l'ADS si que et pots informar. Hi ha l'assamblea anual on s'explica tot" (farmer)

S42-"[…] "La comunicació de les proves als ramaders es bona. Segons quins ramaders se'ls hi reenvia directament el correu amb els resultats. Quan arriben es truca al ramader i se'l informa" (official veterinarian)

S43-"[…] "La comunicación dels resultats de les proves és rápida. Les primeres són in situ i la gama interferó és bastant rápida, entre dos i quatre dies."(farmer)

S44-"[…] "…cuando no nos querían dar los resultados porque como éramos delincuentes. Era el único sitio donde no nos daban los resultados. Porque en teoría sólo marcábamos lo que marcaba el gamma." (private veterinarian)

S45-"[…] Pero si lo fastidioso es que no ves resultados. Entonces la gente está cabreada con eso". (farmer)

S46- "[…] a veces yo creo que tampoco se recogen las muestras como debe ser, luego no se notifica si ha dado el cultivo positivo" (private veterinarian)

iii) Role of wildlife and other domestic reservoirs

S47-"[…] nosotros hemos transmitido primero a la fauna silvestre los espoligotipos de la fauna doméstica y la fauna silvestre nos lo está devolviendo contaminándolos." (official veterinarian)

S48-"Yo lo veo complicadillo eso, porque mientras que no se termine con la fauna salvaje… ¿cómo vas a quitar eso? Tema de venado, tema de jabalíes […] la prueba está que en lo que es vacuno intensivo que no salen de una explotación, que te voy a decir yo, no llega al 2%..." (farmer)

S49-"Con respecto a la fauna salvaje, es muy complicado, porque no puedes…, los animales bueno se contagian por la hierba, por la saliva, por el agua en donde beben…, […] decían que habían inventado un bebedero para que pudieran beber las vacas y no pudieran beber los ciervos, pero al final eso dicen que tampoco ha resultado efectivo" (farmer)

S50-"cuando terminan, se dan la mano, empiezan a salir por la finca coches y se van extendiendo a todos lados. Y a este señor nadie le ha exigido que aquí a la salida haya un vado sanitario que desinfecte los carros, las ruedas. Estos señores que hoy están aquí a lo mejor en la comarca, mañana van a Córdoba y el viernes a Cádiz, y los perros van de aquí a aquí" (official veterinarian)

S51-"Que si una persona tiene dentro de una malla 600 ciervos, no digo que no los tenga, pero que los tenga con el mismo cuidado que la ganadería. Que les haga el saneamiento, porque medios para cogerlos hay". (private veterinarian)

S52-"[…]eso no se controla y se trata en muchos aspectos como la ganadería normal, o sea, se le da de comer como a la ganadería normal, acuden a comer como la ganadería normal, se toca el pito con el coche y se acostumbran los animales a ir a comer" (farmer)

S53-"[…] Explotaciones en las que el ganadero tiene parte de coto de caza… y puedes dedicarla a la actividad cinegética y además es perfectamente comprensible. Lo que pasa es que habrá que ordenar de alguna manera todo esto, ordenarlo y que empiecen a aparecer las ideas oportunas para que esto pueda minimizar las consecuencias que tiene."(official veterinarian)

iv) Risk perception on social aspects

S54-"un ganadero muy problemático que no lo hago yo, lo hace otro y está rodeado por todos los míos. Cayeron todos, y él seguía limpio, hasta que fue una vez la policía judicial y salieron positivas." (private veterinarian)

S55-"[...] Pero verás que yo lo puedo pensar como lo puede pensar muchísimos ganaderos, camiones, coches, personas…, que si está ahí a 20 km puede estar mañana aquí, ¿no?" (farmer)

S56- "Et ve a fer la prova el teu veterinary habilitat i per tant hi tens molta relació del dia a dia i colabora i ajuda en tot el possible" (farmer)

S57-"[...] Luego al final ¿qué pasa?, que encadenas, por no perder al cliente…, pues lo haces. Y así empezó y claro ya no era uno, ya eran varios. Y al final pues algunos nos hemos resistido y yo he perdido muchísimos clientes de bovino" (private veterinarian)

S58-"[...] los ganaderos siempre se han quejado. Algunos decían por aquí que yo tenía un rifle en vez de una jeringa" (private veterinarian).

S59- "A vegades els ramaders busquen algun culpable. Per què han sortit positius? Ells mai tenen la culpa diuen,…., quan hi ha positius la relació a vegades es tensa i es trencadirectament". (private veterinarian)

S60- "...para pasar a T3 esté presente la administración, a ellos les quita un montón de problemas" (official veterinarian)

S61- "La relació entre veterinaris de ADS i oficials és bona. Sempre hi ha persones amb qui no et portes tant bé però en general és bona" (private veterinarian)

S62- "Cap problema amb l'administració. Sempre que ha tingut algun problema ho ha comunicat a l'administració, al Departament i l'han atès bé, ha sortit content i si ells ho han pogut soventar ho han fet" (farmer)

S63- "...el problema es que sí, que por uno, dos o tres que hagan mal o unos cuantos ganaderos, estamos pagando todos" (private veterinarian)

S64- [...] la actitud con la que nos tratan a los ganaderos, en principio nos tratan como si fuéramos delincuentes" (farmer)

S65- "[...] Yo sé de gente que ha saneado y ha llamado por teléfono y "Eh, ¿tienes alguna hinchada?". "No". Eso no puede ser. [...]" (private veterinarian)

S66- "[...] Yo tengo bastante sospecha de que algunas vacas se han leído desde el coche, vamos, que lo dice mucha gente, yo se lo he oído a algún ganadero. Lo hace desde el coche y ¿cómo ves tú que aquello se ha hinchado o no? [...]" (farmer)

S67- "[...] Ese tipo de cosas pues te descorazonan y que hay gente que no hacen las cosas y en estos años terminas enterándote de muchas cosas que han pasado y de gente que les han…, de cosas que no se hacen bien" (farmer)

S68-"[...] y además a todo el mundo por igual, y eso la verdad es que me duele bastante, porque en definitiva esta profesión me parece una profesión bastante digna porque lo que hacemos es producir alimentos para la sociedad y hay que producirlos con calidad, claro."(farmer)

v) Risk perception on bTB and benefits of eradication;

S69-"[...] estamos en un sector, primero muy estratégico y segundo muy miedoso, porque al final la alimentación, lo que las personas nos llevamos a la boca, en el momento en que tienen el más mínimo riesgo no quieren saber nada y simplemente la percepción aunque no sea real, genera unas pérdidas importantísimas" (official veterinarian)

S70- “[…] Hombre, nosotros los veterinarios lo vemos claro, ¿no? El tema de erradicar es un tema de salud pública y de sanidad animal, tienes que acabar con enfermedades más peligrosas y la TB es una de ellas, tenemos que quitarla de en medio a parte por interferencia del mercado, por tema de salud pública, por tema de la propia sanidad animal”. (official veterinarian)

S71- “[…] ¿Qué estamos en una enfermedad que hay que erradicar por supuesto, pero que sin embargo sirve para el consumo humano?” (farmer)

S72- “Si et fan sacrificar 100 animals i 99 van a la cadena humana dius…els han matat perquè han volguts que els matem” (farmer)

S73-“[…] “Eso nunca ha afectado a la producción. Date cuenta que eso se consume por consumo humano, y eso, para mí eso es una cosa que eso cuando se consume no era malo, pero claro que los veterinarios sabrán por lo que será. […]” (farmer)

S74- “[…] Yo no sé exactamente cuál es lo que te puede contagiar, a las personas, porque yo creo que es nada. […] creo que hay un montón de cosas mucho más graves que eso y sin embargo no se les está dando ninguna importancia”. (farmer)

S75-“[…] beneficios si hay porque estar libre de una enfermedad siempre es beneficioso […] las vacas no te van a parir bien si están enfermas, con los becerros exactamente igual”. (farmer)

S76-“[…] yo he tenido animales allí que eran claramente positivos, bueno positivos no, claramente con la enfermedad, y eran animales que tú no podías explotarlos”. (farmer)

S77-“[…] Como realizamos la prueba todo los años, actualmente no tenemos animales realmente enfermos de TBb, lo que tenemos son animales que han estado en contacto con la TBb, no han desarrollado ningún tipo de enfermedad pero como han estado en contacto con la TBb cuando le hacemos la prueba los detectamos como TBb y los sacrificamos, pero el animal desde el punto de vista reproductivo es totalmente rentable.” (official veterinarian)

S78- “[…] “Ellos ven que las dejan circular y nada más, hombre y que está el ganado sano. Pero ellos no ven que eso sea…, es una cosa impuesta y es una cosa que hay que hacer” (private veterinarian)

S79- “[…] Aquí el tema sanitario se lleva un poquito por obligación no porque haya una conciencia… […]” (farmer)

S80- “yo creo que ahí no puede haber duda ninguna de que la erradicación tiene que ser sí o sí, eso es inevitable, porque ya sabemos lo que nos encontramos cuando nos cierran las fronteras…” (farmer)

S81- “[…] tú dime a mí qué hacemos si salen focos, ¿cómo vendes la leche?, ¿cómo vendes la carne? Que los más interesados de que esto no pase, son los ganaderos, aunque son los que más que pierden. […]” (farmer)

S82- “[…] realmente se lo pagamos como un animal enfermo. Ese hecho, pero claro aquí el problema es que si tú de alguna manera subes la indemnizaciones estás primando la enfermedad” (official veterinarian)

S83- “[…] La indemnización te daban, no para comprar una vaca, pero no se perdía tanto, entre la carne y la indemnización pues podías comprar una becerra, pero claro había que sanear. Yo lo veía bien y lo sigo viendo bien”. (farmer)

vi) Future perspective and proposed changes to the programme.

S84- “[…] Erradicar, erradicar, va a ser muy difícil. Pero bajar la prevalencia, sí. Si se implican todos los sectores y se ponen en serio, no ahora sí, ahora no, ahora cambio la legislación porque nos convenga.” (private veterinarian)

S85- *“[…] pero ¿por qué no sacan la vacuna de la TB? ¿Sabes que la hay en humanos? ¿Lo sabías? ¿Te imaginas? Resulta que estamos metidos en un pozo cargándonos cabezas de ganaderos para una cosa que se va a solucionar dentro de 15 o 20 años, sin dar un duro, si se pusieran a poner un duro en 5 años habría un vacuna, ya la hay ¿eh?, de humanos, ¿por qué no la sacan para bóvidos? Se acabó el problema…” (farmer)*

S86- *“[…] Es que estamos viendo la enfermedad como si fuéramos a morirnos al día siguiente como si fueran manzanas envenenadas y a lo mejor hay que admitir que no vamos a superar este problema en poco tiempo, pero no tenemos que cargarnos al ganadero por en medio” (farmer)*
